# Feedback Robust Cubature Kalman Filter for Target Tracking Using an Angle Sensor

**DOI:** 10.3390/s16050629

**Published:** 2016-05-09

**Authors:** Hao Wu, Shuxin Chen, Binfeng Yang, Kun Chen

**Affiliations:** Information and Navigation College Air Force Engineering University, Fenghao east road No.1, Xi’an 710077, China; chenshuxin68@163.com (S.C.); bf_yang@163.com (B.Y.); kunchen365@sina.com (K.C.)

**Keywords:** angle sensor, DOA tracking, nonlinear system, cubature Kalman filter, robust estimation, feedback strategy

## Abstract

The direction of arrival (DOA) tracking problem based on an angle sensor is an important topic in many fields. In this paper, a nonlinear filter named the feedback M-estimation based robust cubature Kalman filter (FMR-CKF) is proposed to deal with measurement outliers from the angle sensor. The filter designs a new equivalent weight function with the Mahalanobis distance to combine the cubature Kalman filter (CKF) with the M-estimation method. Moreover, by embedding a feedback strategy which consists of a splitting and merging procedure, the proper sub-filter (the standard CKF or the robust CKF) can be chosen in each time index. Hence, the probability of the outliers’ misjudgment can be reduced. Numerical experiments show that the FMR-CKF performs better than the CKF and conventional robust filters in terms of accuracy and robustness with good computational efficiency. Additionally, the filter can be extended to the nonlinear applications using other types of sensors.

## 1. Introduction

For the last several decades, the target tracking problem has been the subject of constant and intensive interest in many fields, such as navigation, military applications, and sensors networks [1]. The aim of the target tracking problem is to estimate the position and the velocity of a target from a set of measurements collected by sensors [2]. According to the types of sensors, target tracking can be separated into range-only tracking, direction of arrival (DOA) tracking, and Doppler effects tracking [3,4,5]. Among these methods, DOA tracking is a typical nonlinear problem whose measurements are the basic information from an angle sensor [6,7]. Accordingly, this paper focuses on the DOA tracking with a single angle sensor.

Various nonlinear filtering algorithms have been applied to the DOA tracking problem, such as the extended Kalman filter (EKF) [8], the particle filter (PF) [9,10], the unscented Kalman filter (UKF), and the cubature Kalman filter [11]. The UKF and the CKF are called the deterministically-sampled filters (DSFs) which approximate the Gaussian posterior distribution by means of a set of deterministically sampled points. When the discretization errors of practical systems are not considered, they perform better than the EKF in terms of the accuracy and show better than the PF in terms of the computational cost [11]. However, the UKF and the CKF are effective only under the Gaussian and white assumption of the measurement noise. Actually, measurement outliers are almost inevitable from sensors in practical applications. Owing to their potentially non-Gaussian or non-symmetric properties, measurement outliers may lead to unstable, or even divergent performance. On the other hand, the tracking performance is also concerned with the discretization of the practical system [12]. In this paper, we focus on dealing with outliers, and the discretization errors are not considered.

The hard decision method [13] may prevent outliers to a certain degree. However, it may lead to misjudgment easily owing to the nonlinear property of the DOA tracking. Moreover, once the misjudgment appears, the performance will be degraded significantly. Huber’s M-estimation [14] is an important method to deal with measurement outliers from sensors. It combines the minimum *l*_1_ and *l*_2_ norm estimation technique, which exhibits robustness for contaminated measurements. Unfortunately, the M-estimation is generally limited in linear cases. Indeed, it can be directly extended to nonlinear systems with the help of the first-order Taylor expansion, but the accuracy is difficult to be ensured because of enormous linearized errors in highly nonlinear cases. Recently, combining the DSFs with the M-estimation has been taken into account. They usually reformulate the measurement update stage, and the M-estimation method can be introduced according to a linear or nonlinear regression [15,16,17,18,19]. Nevertheless, the linear or nonlinear regression induces some complex operations such as the matrix inversion and decomposition, which may lead to poor computational efficiency. Moreover, the outliers’ judgment variable in regression-based robust DSFs includes not only measurement errors, but also predicted errors and nonlinear errors in nonlinear systems. Hence the outliers’ judgment threshold is difficult to be determined. Additionally, the robust DSFs do not perform as well as their non-robust versions such that the UKF and the CKF when no outliers appear from sensors, because the weights of the normal measurements may be reduced improperly under Gaussian assumption.

Considering the problems above, the feedback M-estimation based robust cubature Kalman filter (FMR-CKF) is proposed for the single sensor based DOA tracking. Unlike regression-based methods, we introduce the M-estimation to the CKF by means of the equivalent weight function, and the complex computational operations can be avoided. A feedback strategy is also proposed to improve the misjudgment in different cases. When an outlier from the angle sensor is detected, the filter is split into two sub-filters: the standard CKF and the robust CKF. They are simultaneously updated and the sub-filter whose outliers’ judgment variable is smaller at the next time index can be chosen. The FMR-CKF takes full advantage of the accuracy of the CKF and the robustness of the M-estimation and simulation results show that the proposed algorithm performs better than the conventional algorithms whether outliers appear. It should be noted that the proposed filter is different from the gating method in multi-target tracking problem [20,21]. A gating method is used in multi-target tracking problem because the measurement origin is uncertain and it is a binary decision method. On the contrary, the proposed filter is to reduce the influence of the outliers according to the equivalent weight function.

The rest of the paper is organized as follow. Section 2 describes the nonlinear system model of DOA tracking based on an angle sensor. The nonlinear robust filter which is called the MRCKF is presented in Section 3. Section 4 introduces the feedback strategy for DOA tracking. Section 5 yields the performance comparison of the proposed algorithm with conventional algorithms. Conclusions are drawn in Section 6.

## 2. System Model and Cubature Kalman Filter

The target is assumed to be in linear motion with nearly constant velocity for convenience. Of course, the CKF-based method can also handle the condition when the velocity varies. The state vector of the target at time index k (k=1,2,⋯,n) can be given by $x_{k}^{t}\;={\left[x_{k}^{t},y_{k}^{t},{\dot{x}}_{k}^{t},{\dot{y}}_{k}^{t}\right]}^{T}$, where $\left(x_{k}^{t},y_{k}^{t}\right)$ is the position coordinate and $\left({\dot{x}}_{k}^{t},{\dot{y}}_{k}^{t}\right)$ is the velocity coordinate. Similarly, the state vector of the moving angle sensor is expressed by $x_{k}^{o}\;={\left[x_{k}^{o},y_{k}^{o},{\dot{x}}_{k}^{o},{\dot{y}}_{k}^{o}\right]}^{T}$. Thus, the state vector in the relative own-ship reference at time index k is defined as $x_{k}=x_{k}^{t}-x_{k}^{o}={\left[x_{k},y_{k},{\dot{x}}_{k},{\dot{y}}_{k}\right]}^{T}$.

The dynamic model of DOA tracking can be written as:

(1) $$x_{k}=Fx_{k-1}-u_{k-1,k}+v_{k}$$

where F is the transformed matrix, $u_{k-1,k}$ is a vector of deterministic inputs that accounts for the effects of observer accelerations [22], and $v_{k}$ represents the process noise, which is generally assumed to be Gaussian distributed such that N(0,Q). F, $u_{k-1,k}$, and the covariance matrix Q are given by [2]:

$$u_{k-1,k}=\left[\begin{array}{c} x_{k}^{o}-x_{k-1}^{o}-\Delta {\dot{x}}_{k-1}^{o}\\ y_{k}^{o}-y_{k-1}^{o}-\Delta {\dot{y}}_{k-1}^{o}\\ {\dot{x}}_{k}^{o}-{\dot{x}}_{k-1}^{o}\\ {\dot{y}}_{k}^{o}-{\dot{y}}_{k-1}^{o} \end{array}\right],\;F=\left[\begin{array}{cccc} 1 & 0 & \Delta & 0\\ 0 & 1 & 0 & \Delta \\ 0 & 0 & 1 & 0\\ 0 & 0 & 0 & 1 \end{array}\right],\;Q=\left[\begin{array}{cccc} \Delta^{3}/3 & 0 & \Delta^{2}/2 & 0\\ 0 & \Delta^{3}/3 & 0 & \Delta^{2}/2\\ \Delta^{2}/2 & 0 & \Delta & 0\\ 0 & \Delta^{2}/2 & 0 & \Delta \end{array}\right]\cdot q$$

where Δ is the measurement interval and *q* represents the intensity of the process noise.

The measurement model is:

(2) $$z_{k}=h(x_{k})=\arctan\frac{x_{k}}{y_{k}}+w_{k}$$

where $z_{k}$ represents the angle measurement at time index *k* that is obtained by a single angle sensor. $w_{k}$ represents the measurement noise which is generally assumed to be independent and identically Gaussian with a covariance matrix $R_{k}$.

The CKF uses the third-degree spherical-radial rule to numerically approximate the multi-dimensional integral involved in nonlinear Bayesian filtering. The cubature points can be expressed as: $\xi_{j}=\sqrt{n_{x}}{\left[\begin{array}{cc} I_{n_{x}} & -I_{n_{x}} \end{array}\right]}_{j}$, where $I_{n_{x}}$ is the $n_{x}\times n_{x}$ dimensional identity matrix, and ${\left[\cdot \right]}_{j}$ represents the $j^{th}$ column of [⋅] ($j=1,2,\cdots ,2n_{x}$).

At the time update stage, the dynamic model of DOA tracking is typically linear; thus, the mean and associated covariance of the Gaussian predictive density can be directly written as:

(3) $${\hat{x}}_{k|k-1}=F{\hat{x}}_{k-1}-u_{k-1,k}$$

(4) $$P_{k|k-1}=FP_{k-1}F^{T}+Q$$

At the measurement update stage, the predicted covariance is first factorized as $P_{k|k-1}=S_{k|k-1}S_{k|k-1}^{T}$. We then evaluate the cubature points:

(5) $$x_{k|k-1,j}^{*}=S_{k|k-1}\xi_{j}+{\hat{x}}_{k|k-1}$$

The cubature points are propagated through the nonlinear measurement equation:

(6) $$z_{k|k-1,j}^{*}=h(x_{k|k-1,j}^{*})$$

Accordingly, the predicted measurement can be estimated through:

(7) $${\hat{z}}_{k|k-1}=\frac{1}{2n_{x}}\sum_{j=1}^{2n_{x}}z_{k|k-1,j}^{*}$$

The Kalman gain is given by:

(8) $$K_{k}=P_{xz,k|k-1}P_{zz,k|k-1}^{-1}$$

where the innovation covariance matrix $P_{zz,k|k-1}$ and the cross covariance matrix $P_{xz,k|k-1}$ are:

(9) $$P_{zz,k|k-1}=\frac{1}{2n_{x}}\sum_{j=1}^{2n_{x}}\left(z_{k|k-1,j}^{*}-{\hat{z}}_{k|k-1,j}\right){\left(z_{k|k-1,j}^{*}-{\hat{z}}_{k|k-1,j}\right)}^{T}+R_{k}$$

(10) $$P_{xz,k|k-1}=\frac{1}{2n_{x}}\sum_{j=1}^{2n_{x}}\left({\hat{x}}_{k|k-1}-x_{k|k-1,j}^{*}\right){\left({\hat{z}}_{k|k-1}-z_{k|k-1,j}^{*}\right)}^{T}$$

Therefore, the state estimate and the corresponding covariance can be updated as:

(11) $${\hat{x}}_{k|k}={\hat{x}}_{k|k-1}+K_{k}(z_{k}-{\hat{z}}_{k|k-1})$$

(12) $$P_{k|k}=P_{k|k-1}-K_{k}P_{zz,k|k-1}K_{k}^{T}$$

## 3. Robust Cubature Kalman Filter Based on the M-Estimation

Without regard to the discretization errors, the CKF will be accurate and stable if the measurement noise follows Gaussian distribution exactly. However, measurements are easily contaminated by the outliers in practical applications [17]. The outliers will lead to inaccurate performance if we do nothing about them. Accordingly, the contaminated distribution is introduced:

(13) G=(1−ε)D+εM

where D is the dominated distribution, M is the interference distribution, and ε is the contaminated rate. In general, the Gaussian distribution is widely adopted to be the dominated distribution D because it is tractable. The outlier can be regarded as the measurement noise from the interference distribution M. In order to obtain accurate and robust results, both D and M should be considered.

The key of the M-estimation is to keep the weights of normal measurements and to reduce the weights of contaminated measurements. Generally, the weight of the normal measurement is considered the inverse matrix of the measurement covariance such that $p_{k}={\left(R_{k}\right)}^{-1}$. Thus, the innovation covariance matrix of the CKF $P_{zz,k|k-1}$ can be rewritten as:

(14) $$P_{zz,k|k-1}=\frac{1}{2n_{x}}\sum_{j=1}^{2n_{x}}\left(z_{k|k-1,j}^{*}-{\hat{z}}_{k|k-1}\right){\left(z_{k|k-1,j}^{*}-{\hat{z}}_{k|k-1}\right)}^{T}+p_{k}^{-1}$$

The covariance of the outlier which comes from the unknown interference distribution is denoted as ${\bar{R}}_{k}$, and the equivalent weight matrix of the contaminated measurement can be expressed as ${\bar{p}}_{k}={\left({\bar{R}}_{k}\right)}^{-1}$. Consequently, the equivalent innovation covariance matrix ${\bar{P}}_{zz,k|k-1}$ is obtained by:

(15) $${\bar{P}}_{zz,k|k-1}=\frac{1}{2n_{x}}\sum_{j=1}^{2n_{x}}\left(z_{k|k-1,j}^{*}-{\hat{z}}_{k|k-1,j}\right){\left(z_{k|k-1,j}^{*}-{\hat{z}}_{k|k-1,j}\right)}^{T}+{\bar{p}}_{k}^{-1}$$

According to Equation (13), the outliers can be regarded as the data coming from the interference distribution, where the covariance is ${\bar{p}}_{k}^{-1}$.

The corresponding equivalent Kalman gain ${\bar{K}}_{k}$ is given by:

(16) $${\bar{K}}_{k}=P_{xz,k|k-1}{\bar{P}}_{zz,k|k-1}^{-1}$$

Therefore, the estimate state and the corresponding covariance can be updated as:

(17) $${\hat{x}}_{k|k}={\hat{x}}_{k|k-1}+{\bar{K}}_{k}(z_{k}-{\hat{z}}_{k|k-1})$$

(18) $$P_{k|k}=P_{k|k-1}-{\bar{K}}_{k}{\bar{P}}_{zz,k|k-1}{\bar{K}}_{k}^{T}$$

It can be seen from Equations (14) and (15) that the only difference between the CKF and the M-estimation based robust CKF (MRCKF) is the weight matrix, which implies that handling measurement outliers is actually transformed into designing of the equivalent weight matrix ${\bar{p}}_{k}$, *i.e.*, the Huber’s weight function can be expressed as [14]:

(19) $${\bar{p}}_{k}=\left\{ \begin{array}{l} R_{k}^{-1},\left|{\tilde{r}}_{k}\right|\leq c\\ \frac{R_{k}^{-1}\cdot c}{\left|{\tilde{r}}_{k}\right|},\left|{\tilde{r}}_{k}\right|>c \end{array}\right.$$

where ${\tilde{r}}_{k}$ is the standardized residual at time index k and the threshold c is usually chosen as c∈[1.3, 2] [23]. When ${\bar{p}}_{k}=R_{k}^{-1}$, the MRCKF turns into the CKF, which means the CKF is a special case of the MRCKF with ε=0.

It is noted that the outliers’ judgment threshold c is difficult to be determined because the standardized residual includes not only measurement errors, but also predicted errors and nonlinear errors in the DOA tracking problem. In this respect, we consider the standardized innovation:

(20) $$e_{k}=\left(z_{k}-{\hat{z}}_{k|k-1}\right)/S_{zz,k|k-1}$$

where $S_{zz,k|k-1}$ is a square factor of the innovation covariance matrix such that $P_{zz,k|k-1}=S_{zz,k|k-1}S_{zz,k|k-1}^{T}$.

An outliers’ judgment variable can be constructed as:

(21) $$\lambda_{k}=\left(z_{k}-{\hat{z}}_{k|k-1}\right)P_{zz,k|k-1}^{-1}\left(z_{k}-{\hat{z}}_{k|k-1}\right)=e_{k}^{2}$$

$\lambda_{k}$ is the Mahalanobis distance [24] between the actual measurement $z_{k}$ and the predicted measurement ${\hat{z}}_{k|k-1}$ at time index *k*. From a statistical perspective, the Mahalanobis distance provides an inconsistency degree between the actual measurement and the predicted measurement. When the process noise $v_{k-1}$ and the measurement noise $w_{k}$ are both Gaussian, the predicted measurement will be statistically close to the actual measurement. Otherwise, when a measurement outlier appears, $\lambda_{k}$ will become abnormal. It is difficult to verify the real distribution of $e_{k}$ due to nonlinear property of the measurement equation. Here, $e_{k}$ is approximately considered Gaussian and $\lambda_{k}$ will approximately follow Chi-square distribution, such that:

(22) $$\lambda_{k}=\chi^{2}\left(1\right)$$

where $\chi^{2}\left(m\right)$ represents the Chi-square distribution with *m* degrees of freedom.

Denoting the significance level as α, we obtain:

(23) $$p\left(\lambda_{k}>\gamma_{\alpha }\right)=1-\alpha$$

where p(⋅) is probabilistic computation operator and $\gamma_{\alpha }$ represents the α-quantile.

When a measurement outlier appears, $\lambda_{k}$ will generally deviate from Chi-square distribution. In other words, if $\lambda_{k}>\gamma_{\alpha }$ and 1−α is chosen as a small value, the measurement can be regarded as an abnormal measurement which is contaminated by the outlier. Consequently, the outliers’ judgment threshold $\gamma_{\alpha }$ can be chosen according to the significance level α from Chi-square distribution tables.

When the filter is modified by the equivalent weight, the new judgment variable should be not exceeded $\gamma_{\alpha }$, otherwise the robust method will be failure. *i.e.*:

(24) $${\bar{\lambda }}_{k}=\left(z_{k}-{\hat{z}}_{k|k-1}\right){\bar{P}}_{zz,k|k-1}^{-1}\left(z_{k}-{\hat{z}}_{k|k-1}\right)\leq \gamma_{\alpha }$$

Substituting Equations (14) and (15) into Equation (24), we have:

(25) $${\bar{p}}_{k}^{-1}\geq \frac{\lambda_{k}P_{zz,k|k-1}}{\gamma_{\alpha }}-P_{zz,k|k-1}+R_{k}$$

Therefore, a new equivalent weight function can be described as:

(26) $${\bar{p}}_{k}=\left\{ \begin{array}{cc} R_{k}^{-1}, & \lambda_{k}\leq \gamma_{\alpha }\\ {\left(\frac{\lambda_{k}P_{zz,k|k-1}}{\gamma_{\alpha }}-P_{zz,k|k-1}+R_{k}\right)}^{-1}, & \lambda_{k}>\gamma_{\alpha }\; \end{array}\right.$$

Compared with Equation (19), the outliers’ judgment threshold of Equation (26) is determined naturally according to confidence level of $\lambda_{k}$, and improper experiential value can be avoided. From a computational complexity perspective, iterations and regression procedure are not required, which implies that the MRCKF shows better than the regression-based robust DSFs in terms of the computational efficiency. Although the M-estimation is introduced to the CKF based on the DOA problem, the MRCKF can be extended to other nonlinear filtering system.

## 4. Feedback Strategy for DOA Tracking

If there are no measurement outliers from the angle sensor (the measurement noise follows the Gaussian distribution exactly), the performance of the CKF is generally better than that of its robust versions (such as the MRCKF). This is because the MRCKF do not always minimize the $l_{2}$ norm in that case, and the weights of normal measurements may be reduced improper during the filtering update. What is worse, nonlinear property of the DOA tracking system would make $\lambda_{k}$ deviate from Chi-square distribution, which further increases the misjudgment rate. Of course, increasing the number of the measurements in each time index may improve the problem above. Unfortunately, there is only one measurement in the DOA tracking problem at each time index, which implies that the DOA filters are more sensitive to the measurement outliers and the misjudgment occurs easily.

**Figure 1:**
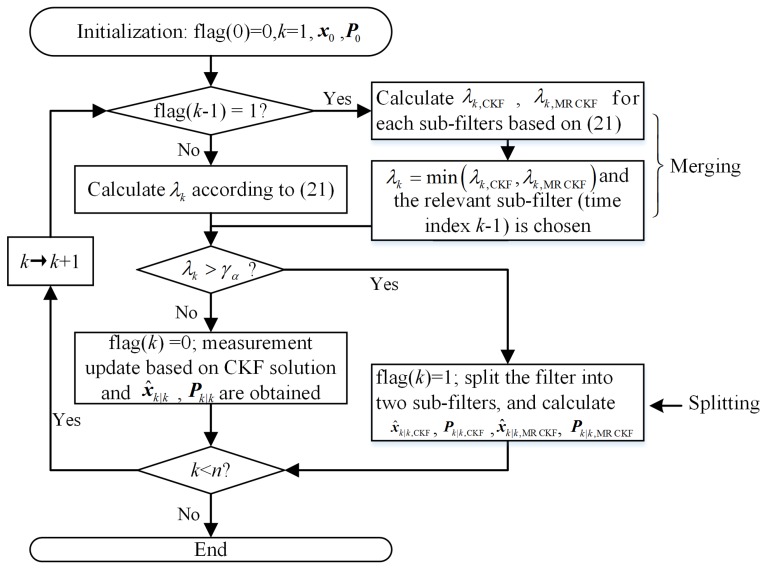
The flow diagram of the FMR-CKF.

Here, we do not consider continuous outliers and propose a feedback strategy to solve the problem. When the outliers’ judgment variable $\lambda_{k}$ exceeds the threshold at time index *k*, the filter is split into two sub-filters: the standard CKF and the MRCKF. Then they are updated according to their solution frameworks. At time index *k* + 1, the outliers’ judgment variables of the sub-filters can be obtained. If $\lambda_{k+1,MRCKF}<\lambda_{k+1,CKF}$, the MRCKF is chosen, otherwise the CKF is chosen. The new filtering algorithm with the feedback strategy is named the feedback MRCKF (FMR-CKF). The flow diagram of the FMR-CKF can be summarized in Figure 1.

Smaller $\lambda_{k+1}$ means the predicted information which is obtained according to the corresponding sub-filter at time index *k* is closer to the actual measurement at time index *k* + 1. That is, the sub-filter with a smaller $\lambda_{k+1}$ performs better at time index *k*. The better sub-filter can be chosen according to the judgment variables at the next time index. Meanwhile, the probability of misjudgment can be reduced.

## 5. Numerical Experiments

The algorithms are compared in this section. System model is described as Equations (1) and (2). We assume that the measurement interval Δ = 1 min and the process noise intensity *q* = 10^−10^ km^2^/s^3^. To satisfy the target observability requirements, a single observer is assumed to be maneuvering where the start coordinate is (0,0). From 0 min to 12 min, the observer is in uniform linear motion, where the speed is 4 knots and the course is 140°. From 13 min to 17 min, the observer is in uniform turning motion, where the speed is 4 knots and the turn rate is 30°. From 18 min to 30 min, the observer is in uniform linear motion, where the speed is 4 knots and the course is 290°.

The initialization method is the same as [2,25]. For fair comparison, the same initial condition are set for different algorithms. The initial range from the target to the angle senor *r* = 5 km and the estimated standard deviation σ*_r_* = 1.5 km. The standard deviation of the angle estimate σ*_z_* = 1°; Similarly, the initial velocity of the target *s* = 4 knots and the estimated standard deviation σ*_s_* = 2 knots. The true target course *c* = −120°, and the estimated standard deviation σ*_c_* = π/$\sqrt{12}$. The initial state and covariance of the algorithms can be estimated according to [2]. In the nonlinear regression based novel robust UKF (NRUKF), the scale parameter *κ* = 0 and the other parameters are initialized according to [17]. In the MRCKF and FMR-CKF, α = 95% and *Υ_a_* is 3.841.

The root mean square error in position (RMSE_pos_) at time index , and the mean squared error in position (MSE_pos_) for each Monte Carlo runs are, respectively, defined as:

(27) $${\text{RMSE}}_{\text{pos},k}=\sqrt{\frac{1}{L}\sum_{i=1}^{L}{\left({\hat{x}}_{k,i}^{t}-x_{k,i}^{t}\right)}^{2}+{\left({\hat{y}}_{k,i}^{t}-y_{k,i}^{t}\right)}^{2}}$$

(28) $${\text{MSE}}_{\text{pos},i}=\frac{1}{n}\sum_{k=1}^{n}\left[{\left(x_{k}^{t}-{\hat{x}}_{k|k,i}^{t}\right)}^{2}+{\left(y_{k}^{t}-{\hat{y}}_{k|k,i}^{t}\right)}^{2}\right]$$

where *L* = 500 is Monte-Carlo times and i=1,2,⋯,L.

Two cases are considered for illustrating the performance of the algorithms.

**Case 1:** No outliers in angle measurements. The measurement noise follows Gaussian distribution exactly, and contaminated rate ε=0.

**Case 2:** The dominated distribution is Gaussian. The outliers exist at 20 min, 25 min, 30 min, and the true values of the outliers are $6\sigma_{z}$. A large outlier exists at 35 min, and the true value is $30\sigma_{z}$.

**Figure 2:**
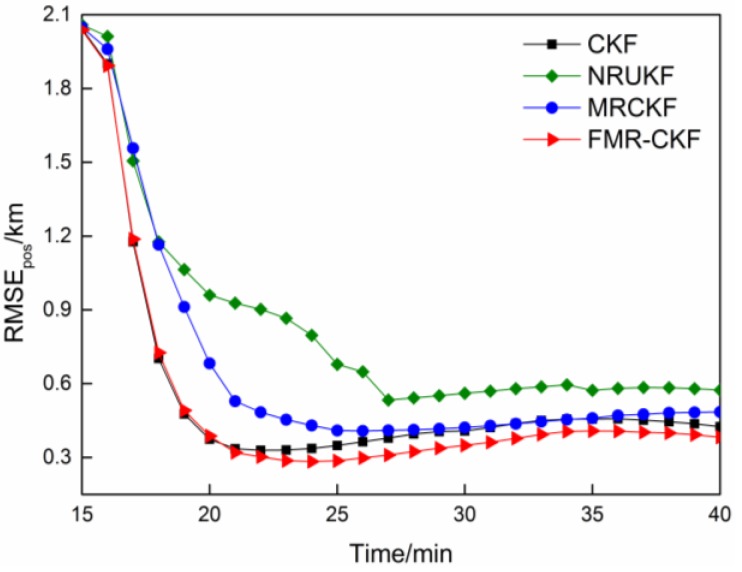
The RMSE_pos_ comparison when there are no outliers.

**Figure 3:**
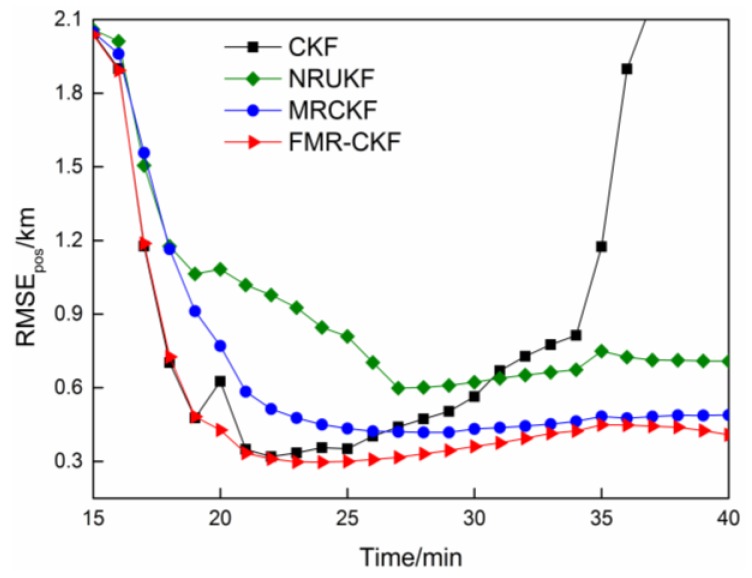
The RMSE_pos_ comparison when outliers appear.

The RMSE_pos_ of the various filtering algorithms in Case 1 and Case 2 are described in Figure 2 and Figure 3, respectively.

The EKF and gating rule-based method often fails to yield meaningful results. Hence, we do not present the results of the EKF and the gating rule-based method. As shown in Figure 2, when the measurement noise follows Gaussian distribution exactly, the NRUKF and the MRCKF is inferior to that of the CKF, because the normal measurement errors may be misjudged as the outliers in the NRUKF and the MRCKF under Gaussian assumption. On the contrary, the FMR-CKF outperforms the CKF because the FMR-CKF can select the better sub-filter (the CKF or the MRCKF) according to the feedback strategy in each time index. For instance, when a large measurement error appears (follows Gaussian distribution), the FMR-CKF selects the MRCKF to reduce the influence of the large measurement error, but the CKF neglects it and then the performance is degraded. Hence the FMR-CKF performs the best in this case.

As shown in Figure 3, when measurement outliers appear, the results of the CKF become unreliable due to non-robust property. Among the robust filters, the MRCKF outperforms the NRUKF because the MRCKF introduces the M-estimation without linearization and approximate regression procedures, which makes better use of the accuracy of the CKF and the robustness of the M-estimation. Meanwhile, the outliers’ judgment threshold of the MRCKF is naturally obtained according to Chi-square distribution, but that of the NRUKF is generally experiential, which may lead to more misjudgments. Compared with the MRCKF, the FMR-CKF further avoids the misjudgment based on the feedback strategy and, hence, it performs better than the MRCKF.

**Figure 4:**
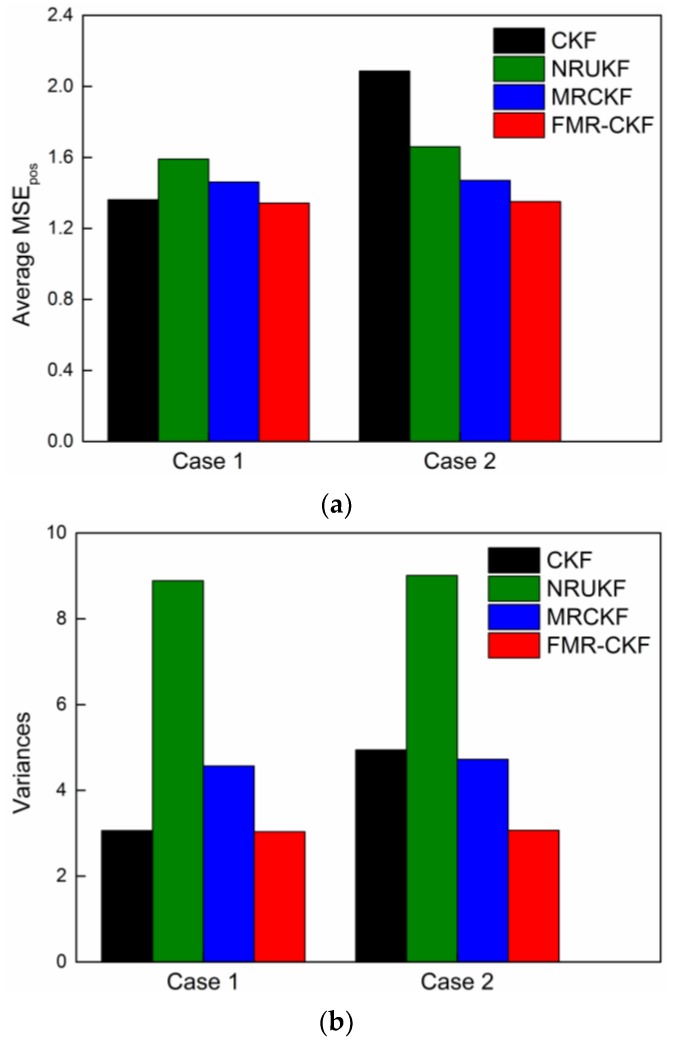
The MSE_pos_ comparison of different filtering algorithms. (**a**) The average MSEs in position; and (**b**)the corresponding variances.

The average MSEs in position of the filters are shown in Figure 4a and the corresponding variances are shown in Figure 4b.

As can be seen in Figure 4, the average MSEs_pos_ and the corresponding variances of the FMR-CKF change smaller in different cases and are obviously lower than those of the other algorithms. It demonstrates that the FMR-CKF is more numerically stable and consistent than the other algorithms. The judgment variable of the NRUKF, *i.e., the* measurement residual, includes both predicted error and nonlinear error, which leads to unstable performance and, hence, the variances of the NRUKF are significantly higher than those of the other algorithms in different cases.

To compare the computational cost, the computational time of the algorithms investigated relative to that of the CKF in Case 2 are shown [2]. Of course, some errors may exist because coding of each method may not be equivalently optimized in the simulation, but it can be seen that FMR-CKF significantly outperforms the NRUKF in terms of the computational time.

Table 1 shows that the NRUKF runs much slower than the other existing algorithms as it requires iterations and complex operations, such as matrix decomposition and inversion in the regression procedure. The FMR-CKF introduces computational time in the equivalent weight computation and the splitting procedure, which shows a major computational advantage over the NRUKF.

## 6. Conclusions

We have proposed the FMR-CKF to deal with the DOA tracking problem when measurement outliers appear from the angle sensor. Compared with the DSFs such as the CKF and UKF, the FMR-CKF can solve measurement outliers effectively. Compared with the regression-based robust DSFs, the new filter takes fully advantage of the robustness of the M-estimation and the accuracy of the CKF by means of the equivalent weight function and the feedback strategy. Additionally, the CKF solution framework is retained, which implies that the FMR-CKF is a derivative-free and computationally-efficient filter. Simulation results show that the proposed algorithm exhibits the best performance (both in filter accuracy and stability) whether the measurement outliers appear. While the example was the 2-dimensional DOA problem in this paper, the FMR-CKF has potential applications in higher dimensional problems or other nonlinear situations.

## Figures and Tables

**Table 1:** Relative computation times of the algorithms.

	CKF	NRUKF	MRCKF	FMR-CKF
Relative computation times	1	2.41	1.13	1.32
